# Restoration of the ER stress response protein TDAG51 in hepatocytes mitigates NAFLD in mice

**DOI:** 10.1016/j.jbc.2024.105655

**Published:** 2024-01-16

**Authors:** Tamana R. Yousof, Celeste C. Bouchard, Mihnea Alb, Edward G. Lynn, Sárka Lhoták, Hua Jiang, Melissa MacDonald, Hui Li, Jae H. Byun, Yumna Makda, Maria Athanasopoulos, Kenneth N. Maclean, Nathan J. Cherrington, Asghar Naqvi, Suleiman A. Igdoura, Joan C. Krepinsky, Gregory R. Steinberg, Richard C. Austin

**Affiliations:** 1 Division of Nephrology, Department of Medicine, The Research Institute of St. Joe's Hamilton and the Hamilton Centre for Kidney Research, McMaster University, Hamilton, Ontario, Canada; 2 Department of Pediatrics, School of Medicine, University of Colorado Health Sciences Center, Aurora, Colorado, USA; 3 Department of Pharmacology and Toxicology, University of Arizona, Tucson, Arizona, USA; 4 Department of Biology, McMaster University, Hamilton, Ontario, Canada; 5 Department of Pathology and Molecular Medicine, St. Joseph's Healthcare Hamilton, McMaster University, Hamilton, Ontario, Canada; 6 Department of Pathology and Molecular Medicine, McMaster University, Hamilton, Ontario, Canada; 7 Centre for Metabolism, Obesity and Diabetes Research, McMaster University, Hamilton, Ontario, Canada; 8 Division of Endocrinology, Department of Medicine, McMaster University, Hamilton, Ontario, Canada

**Keywords:** liver, hepatocyte, insulin resistance, lipid metabolism, triglyceride, obesity

## Abstract

Endoplasmic reticulum stress is associated with insulin resistance and the development of nonalcoholic fatty liver disease. Deficiency of the endoplasmic reticulum stress response T-cell death–associated gene 51 (TDAG51) (*TDAG51*^*−/−*^) in mice promotes the development of high-fat diet (HFD)-induced obesity, fatty liver, and hepatic insulin resistance. However, whether this effect is due specifically to hepatic TDAG51 deficiency is unknown. Here, we report that hepatic TDAG51 protein levels are consistently reduced in multiple mouse models of liver steatosis and injury as well as in liver biopsies from patients with liver disease compared to normal controls. Delivery of a liver-specific adeno-associated virus (AAV) increased hepatic expression of a TDAG51-GFP fusion protein in WT, *TDAG51*^*−/−*^, and leptin-deficient (*ob/ob*) mice. Restoration of hepatic TDAG51 protein was sufficient to increase insulin sensitivity while reducing body weight and fatty liver in HFD fed *TDAG51*^*−/−*^ mice and in *ob/ob* mice. *TDAG51*^*−/−*^ mice expressing ectopic TDAG51 display improved Akt (Ser473) phosphorylation, post-insulin stimulation. HFD-fed *TDAG51*^*−/−*^ mice treated with AAV-TDAG51-GFP displayed reduced lipogenic gene expression, increased beta-oxidation and lowered hepatic and serum triglycerides, findings consistent with reduced liver weight. Further, AAV-TDAG51-GFP–treated *TDAG51*^*−/−*^ mice exhibited reduced hepatic precursor and cleaved sterol regulatory–element binding proteins (SREBP-1 and SREBP-2). *In vitro* studies confirmed the lipid-lowering effect of TDAG51 overexpression in oleic acid–treated Huh7 cells. These studies suggest that maintaining hepatic TDAG51 protein levels represents a viable therapeutic approach for the treatment of obesity and insulin resistance associated with nonalcoholic fatty liver disease.

The global obesity epidemic is associated with the rise in the development of insulin resistance (IR) and nonalcoholic fatty liver disease (NAFLD) (1). NAFLD is defined as the accumulation of fat in the liver and is involved in a spectrum of pathological changes which can progress to nonalcoholic steatohepatitis (NASH), cirrhosis, and hepatocellular carcinoma (2, 3, 4). The progression of NAFLD pathogenesis is largely unknown; however, a state of imbalance between the uptake, synthesis, oxidation, and export of fatty acids by the liver has been established (5). The accumulation of lipids, a hallmark of NAFLD, subsequently leads to cellular stress and hepatic injury. Steatotic livers often present with IR and are associated with perturbed endoplasmic reticulum (ER) proteostasis in hepatocytes (6, 7, 8). Upon ER stress, the unfolded protein response is an adaptive signaling pathway activated in an attempt to restore ER proteostasis.

The ER-responsive gene, T-cell death–associated gene 51 (TDAG51), a homolog of the human Pleckstrin homology–like domain A-1 (PHLDA-1) gene, is a regulator of protein synthesis downstream of the PERK pathway (9). Genetic ablation of TDAG51 in mice (*TDAG51*^−/−^) promotes IR, greater steatosis and late-onset obesity compared to age-matched WT controls (10). Though the cell-specific metabolic effects of TDAG51 are only partially understood, hepatocyte-specific adenovirus-delivered shRNA knockdown of TDAG51 was associated with increased lipid droplet size in chow-fed mice (11). These studies suggest that diminished levels of hepatic TDAG51 protein are positively associated with obesity, hepatic steatosis, and IR. In support of this concept, well-established mouse models of obesity, including high-fat diet (HFD)-fed WT mice and mice lacking the satiety hormone, leptin, demonstrate marked reduction in hepatic TDAG51 protein levels (10). In this study, we further characterize the loss of TDAG51 protein levels in several mouse models of liver injury independent of lipid accumulation. Herein, this study establishes the relevance of TDAG51/PHLDA1 protein levels in human liver disease. Although metabolic studies have been employed in mice lacking TDAG51 in all cell types and tissues, the purpose of this study was to directly investigate the potential role of enhancing hepatocyte-specific TDAG51 levels, its impact on extrahepatic tissues and related outcomes in the context of obesity and IR.

We report here that TDAG51 is reduced in several distinct liver injury models and that restoring hepatic TDAG51 levels rescues the underlying IR and NAFLD in diet- and genetic-induced mouse models of fatty liver disease. Hepatic TDAG51 restoration significantly reduces adipose tissue weight, suggesting that TDAG51 exerts its effects *via* regulatory crosstalk in tissues where TDAG51 is not exogenously expressed. These findings suggest that restoring hepatic TDAG51 may serve as a treatment strategy for reducing obesity and concomitant fatty liver disease.

## Results

### Hepatic TDAG51 protein levels are significantly reduced in numerous mouse models of NAFLD and in human NASH

**Figure 1:**
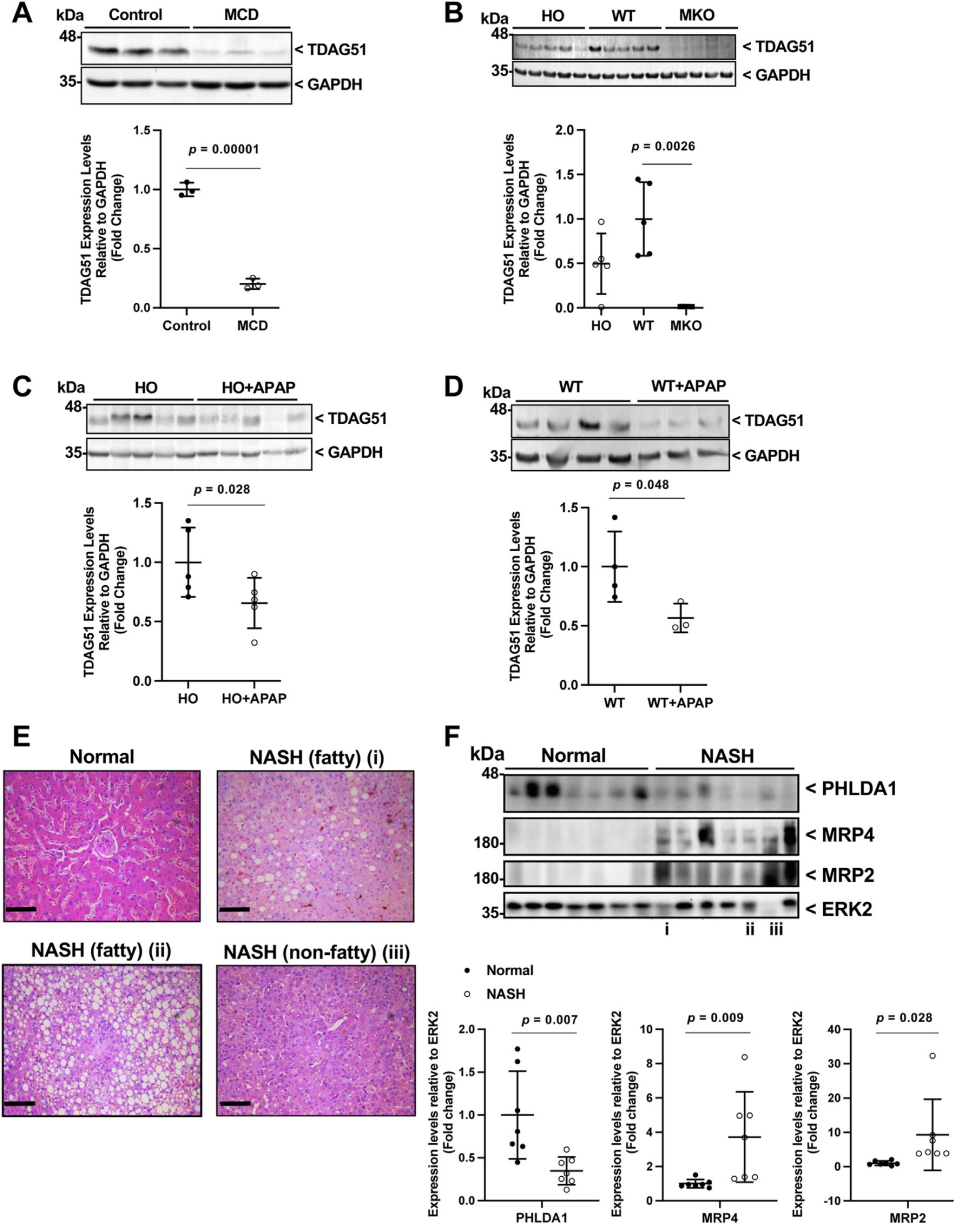
**Hepatic TDAG51 protein expression is significantly reduced inhuman NASH andmultiple mouse models of liver injury or steatosis.** Representative immunoblot and densitometry of TDAG51 protein levels relative to GAPDH protein in the livers of *A*, WT mice on an 18-day normal chow or methionine-choline–deficient (MCD) diet (n = 8), *B*, WT, human-only *cystathionine-beta-synthase* (HO), and *cystathionine-beta-synthase*-null also known as Maeda KO (MKO) mice (n = 8), (*C*) HO mice treated with acetaminophen (APAP) compared to saline-treated controls (n = 8), (*D*) WT mice treated with APAP compared to saline-treated controls (n = 8). *E*, H&E-stained sections of human liver. Histologic assessment of the livers was determined using light microscopy at 20× magnification. Sections for normal, NASH with steatosis (*i, ii*), and NASH without steatosis (*iii*) correspond to the same liver lysates immunoblotted in Figure 1*F*. *F*, representative immunoblots and densitometry of human liver lysates from normal and NASH patients probed for PHLDA1 (human homolog of mouse TDAG51), and NASH markers, MRP2 and MRP4, relative to ERK2 as a loading control. Statistical comparisons were assessed with an independent two-tailed Student’s *t* tests for TDAG51/PHLDA1 densitometry and increased levels of MRP2 and MRP4 were assessed with independent one-tailed Student’s t tests. Data are represented as means with error bars representing SD. *A*, ∗∗∗∗*p* = 0.00001 *versus* control. *B*, ∗∗*p* = 0.0026 *versus* WT. *C*, ∗*p* = 0.028 *versus* HO. *D*, ∗*p* = 0.048 *versus* WT. *F*, ∗∗*p* < 0.007 *versus* normal, ∗∗*p* < 0.009 *versus* normal, and ∗*p* = 0.028 *versus* normal. Scale bar represents 100 μm. AAV, adeno-associated virus; ERK2, extracellular signal-regulated kinase 2; MRP, multidrug resistance–associated protein; NASH, nonalcoholic steatohepatitis; PHLDA, Pleckstrin homology–like domain A-1; TDAG51, T-cell death–associated gene 51.

*TDAG51*^*−/−*^ mice develop mature-onset obesity, hepatic steatosis, and IR when fed a chow diet (10). Furthermore, hepatic TDAG51 protein was markedly decreased in both diet- and genetic-induced mouse models of fatty liver disease (10). Given the complexity of these obesity models and known correlations between obesity, IR, and NAFLD, we examined the expression of TDAG51 in NAFLD in the absence of obesity and IR. We first examined male C57BL/6J mice on a methionine-choline deficient diet; methionine-choline deficiency causes rapid steatosis, but not IR or obesity (12), by impairing VLDL secretion due to oxidative stress and lack of lipotropes (13, 14). These mice exhibited significant reductions in hepatic TDAG51 protein compared to controls (Fig. 1*A*). The second model studied was the Maeda KO (MKO) mouse model. MKO mice are ablated for the cystathionine β-synthase (*CBS*) gene, the first enzyme in the *trans-*sulfuration pathway that catalyzes the condensation of serine and homocysteine to cystathionine (14, 15). Ninety percent of MKO mice die from liver failure in the first 2 weeks of life and mice that live beyond 2 weeks have severe steatosis and fibrosis (15). MKO mice have almost undetectable hepatic TDAG51 protein levels in comparison to WT mice (Fig. 1*B*). MKO mice with a transgenic copy of the human *CBS* gene under the control of a human promoter (HO) were then examined for hepatic TDAG51 protein expression (16). HO mice express approximately 1% of normal levels of CBS, which can partially reverse the severe steatohepatitis phenotype seen in the *CBS*-null mice. Compared to MKO mice, HO mice have significantly higher TDAG51 protein levels (Fig. 1*B*). HO mice administered a toxic dose of acetaminophen (APAP), a compound that is well-known to induce liver injury in rodents (17) and humans (18), progresses to necrosis rather than steatosis. These APAP-treated HO mice exhibit further liver injury in comparison to untreated HO mice (Fig. 1*C*). Correspondingly, TDAG51 protein levels were significantly lower in mice treated with APAP than controls (Fig. 1, *C* and *D*). In the models described above, hepatic TDAG51 protein levels were markedly decreased, while hepatic TDAG51 mRNA expression remained unchanged (data not shown). Collectively, these findings suggest post-translational degradation of hepatic TDAG51 protein is associated with various models of liver steatosis and injury, independent of obesity and IR.

In addition to these mouse models of hepatic injury and/or steatosis, human liver lysates were obtained from patients with NASH, defined as the presence of hepatocyte injury, inflammatory infiltrates, and/or collagen deposition (4). Histology of human liver biopsies were classified by NASH fatty (Fig. 1*E* *i* & *ii*) diagnosed as >5% fatty infiltration with macrovesicular steatosis or NASH-nonfatty (Fig. 1*E* *iii*) diagnosed as <5% fatty infiltration with significant inflammation and fibrosis (19). Markers associated with NASH progression, multidrug resistance–associated proteins 2 and 4 (MRP2 and MRP4), were elevated in NASH samples when normalized to extracellular signal-regulated kinase 2 (ERK2) (Fig. 1*F*), as described previously (19, 20, 21). Hepatic PHLDA1 protein level was significantly reduced in human NASH patients compared to normal controls.

### AAV expression of GFP or TDAG51-GFP fusion protein is liver-specific

**Figure 2:**
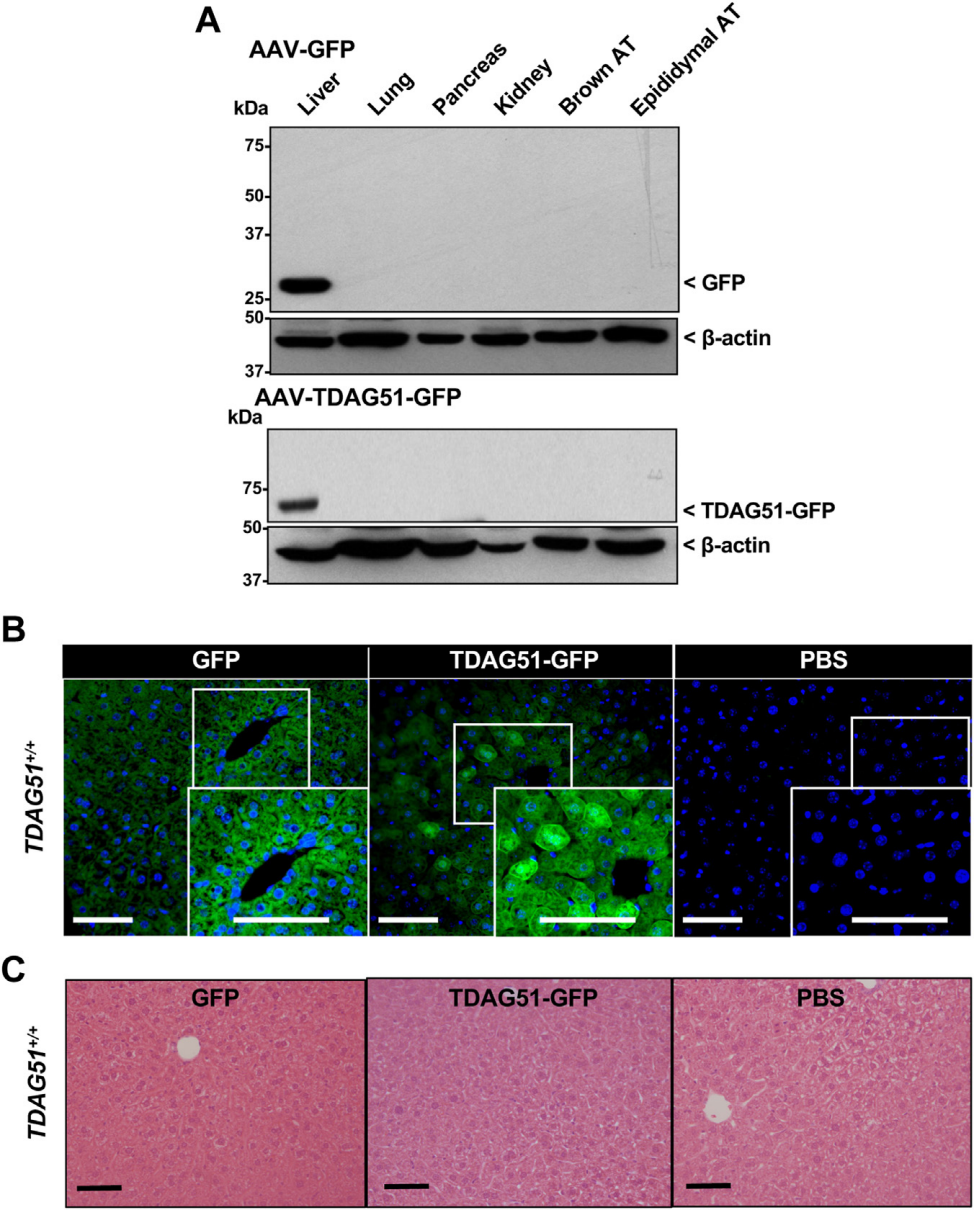
**Liver-specific expression of GFP or TDAG51-GFP fusion protein in chow-fed mice.** WT C57BL/6J mice 8 weeks of age were injected with AAV encoding either GFP or TDAG51-GFP. After 4 weeks post-injection, tissues were immunoblotted for GFP or TDAG51-GFP. *A*, representative immunoblots for GFP (27-kDa) or TDAG51-GFP (67-kDa) using an anti-GFP antibody. Immunoblots were reprobed for β-actin as a loading control. Both GFP and TDAG51-GFP protein were exclusively expressed in liver compared to other tissues. *B*, GFP fluorescent images of liver sections from WT C57BL/6J mice 4 weeks postinjection with 5 × 10^11^ AAV-GFP, 5 × 10^11^ AAV-TDAG51-GFP genome containing particles or PBS. *Green* represents GFP while *blue* represents 4′,6-diamidino-2-phenylindole (nuclei). *C*, representative images of H&E-stained livers 4 weeks post-injection (*bottom panel*). N = 6 mice per group. The scale bar represents 10 μm. AAV, adeno-associated virus; HFD, high-fat diet; TDAG51, T-cell death–associated gene 51.

Ectopic expression of GFP or TDAG51-GFP fusion protein in the liver was initially assessed in 9-week-old C57BL/6J WT mice injected with adeno-associated virus (AAV) serotype 8 encoding either GFP alone or a TDAG51-GFP construct driven by an albumin promoter (Fig. 2). At 4 weeks post-injection of AAV particles encoding GFP, immunoblot analysis using an anti-GFP antibody revealed the expected 28-kDa GFP protein expressed exclusively in the livers of chow-fed WT mice (Fig. 2*A*, *top panel*). Similarly, at 4 weeks post-injection of AAV particles encoding TDAG51-GFP, immunoblot analysis using an anti-GFP antibody revealed expression of the expected 68-kDa TDAG51-GFP fusion protein exclusively in the liver of chow-fed WT mice (Fig. 2*A, bottom panel*). In addition, anti-TDAG51 antibodies also recognized the expressed 68 kDa TDAG51-GFP fusion protein in the livers of AAV-TDAG51-GFP–injected mice (Fig. S1). These findings confirm that overexpression of GFP or the TDAG51-GFP fusion protein using this AAV vector was liver-specific. Immunofluorescent staining of hepatocytes in liver sections from AAV-GFP injected WT mice showed that GFP expression was predominantly cytosolic with some cell membrane and nuclear staining, 4 weeks post-injection (Fig. 2*B*). Consistent with previous studies (10), immunofluorescent staining of hepatocytes from mouse liver sections showed that the TDAG51-GFP fusion protein was predominantly found in the cytoplasm and plasma membrane (Fig. 2*B*). In contrast, GFP immunofluorescence was undetectable in livers of PBS-injected WT mice. H&E staining of the liver sections showed no detectable histological differences between the livers from WT mice treated with AAV-GFP, AAV-TDAG51-GFP, or PBS (Fig. 2*C*). Taken together, these findings demonstrate liver-specific expression of TDAG51 in chow-fed WT mice and its cellular localization is consistent with previous findings (10).

### Restoring hepatic TDAG51 expression in TDAG51−/− mice improves insulin signaling and significantly reduces body and liver weight

**Figure 3:**
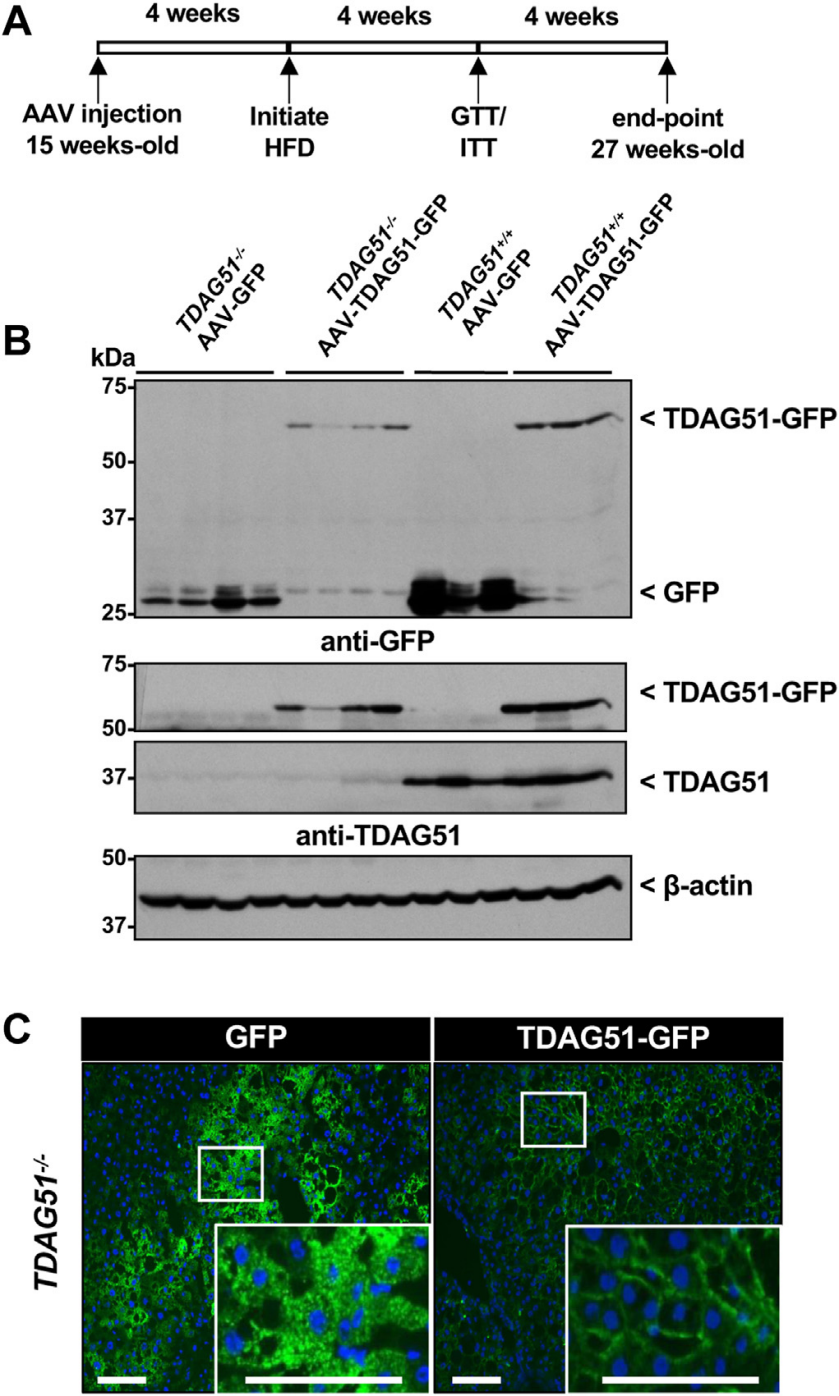
**Expression of hepatic TDAG51 AAV in *TDAG51***^***−/−***^**mice.***A*, fifteen-week-old *TDAG51*^−/−^ mice were injected with 5 × 10^11^ genome containing particles of AAV-GFP or AAV-TDAG51-GFP. Four weeks after AAV injection, mice were fed HFD and glucose tolerance (GTT) and insulin tolerance tests (ITT) were performed 4 weeks later (8 weeks postinjection). To assess hepatic TDAG51-GFP protein levels, mice were sacrificed at 27 weeks old, and the livers harvested, snap-frozen, and total liver lysates immunoblotted for TDAG51 or GFP. *B*, representative immunoblots for TDAG51 and GFP from livers of *TDAG51*^−/−^ mice injected with AAV-GFP or AAV-TDAG51-GFP compared to WT controls. The 68-kDa TDAG51-GFP fusion protein was detected by both the anti-GFP and anti-TDAG51 antibody. Hepatic expression of the TDAG51-GFP fusion protein was reduced in the livers of TDAG51^−/−^ mice compared to WT. *C*, representative GFP fluorescent images of liver sections from TDAG51^−/−^ mice fed HFD. *Green* represents GFP, and *blue* represents 4′,6-diamidino-2-phenylindole (nuclei). N = 3 to 4 mice per group. The scale bar represents 10 μm. AAV, adeno-associated virus; HFD, high-fat diet; TDAG51, T-cell death–associated gene 51.

*TDAG51*^*−/−*^ mice at 15 weeks of age were injected with either AAV-GFP or AAV-TDAG51-GFP and fed a HFD 4 weeks post-injection. Insulin and glucose tolerance tests (GTTs) were subsequently performed 4 weeks post-HFD feeding (Fig. 3*A*). At 27 weeks of age, livers were harvested and ectopic hepatic GFP and TDAG51-GFP protein expression was detected by immunoblotting (Fig. 3*B*). GFP protein expression was observed in both WT and *TDAG51*^*−/−*^ mice injected with AAV-GFP. Furthermore, TDAG51-GFP fusion protein expression was found in liver lysates immunoblotted against antibodies to either TDAG51 or GFP. Ectopic TDAG51-GFP expression was observed after 12 weeks post-injection in TDAG51^*−/−*^ mice but was significantly reduced by approximately 56% compared to WT mice (*p* < 0.02) (Fig. 3*B*). As expected, endogenous TDAG51 protein (40-kDa) was detected only in WT mice and that increased ectopic expression of TDAG51-GFP did not significantly alter endogenous TDAG51 levels. Similarly, immunofluorescence staining of liver sections from *TDAG51*^*−/−*^ mice detected GFP or TDAG51-GFP protein in hepatocytes (Fig. 3*C*). Most of the hepatic TDAG51-GFP expression localized to the plasma membrane, whereas GFP expression was cytosolic in AAV-injected *TDAG51*^*−/−*^ mice. However, the intensity of hepatic GFP expression was much higher than the GFP-TDAG51 fusion protein in *TDAG51*^*−/−*^ mice, corresponding with the immunoblots in Figure 3*B*. These findings suggest a decrease in the stability of TDAG51-GFP and, like endogenous TDAG51, may be subject to proteolytic degradation.

**Figure 4:**
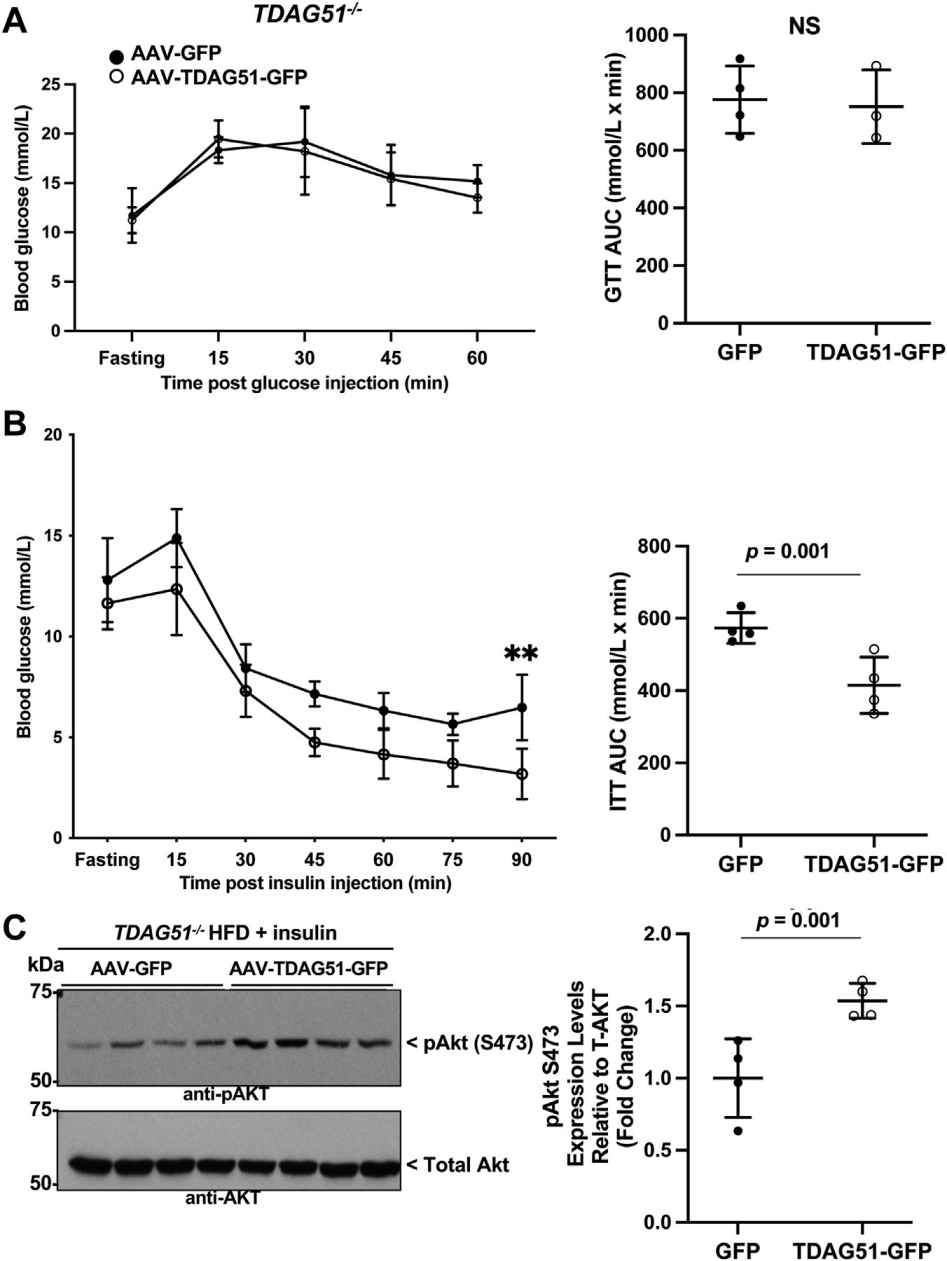
**Restoration of hepatic TDAG51 protein improves insulin sensitivity in *TDAG51***^***−/−***^**mice fed HFD.***A*, glucose tolerance test (GTT) of *TDAG51*^*−/−*^ mice after 4 weeks on HFD. Data represent the mean ± SD. Groups were compared by two-way ANOVA using repeated measures (time × AAV: *F* = 0.68, *p* > 0.05; time: *F* = 24.63, *p* < 0.0001; AAV: *F* = 0.08, *p* > 0.05). Area under the curve (AUC) is represented in the *right panel*; groups were compared by student’s independent *t* test (two-tailed). Data represent the mean ± SD. *B*, insulin tolerance test (ITT) of *TDAG51*^*−/−*^ mice after 4 weeks on HFD. Data represent the mean ± SD. Groups were compared by two-way ANOVA using repeated measures (time × AAV: *F* = 1.1, *p* > 0.05; time: *F* = 99.7, *p* < 0.0001; AAV: *F* = 5.5, *p* < 0.02). Sidak’s multiple comparison used post hoc (90 min, ∗∗*p* < 0.01). Area under the curve (AUC) is represented in the *right panel*; groups were compared by student’s independent *t* test (two-tailed). Data represent the mean ± SD (∗∗*p* = 0.01). *C*, fourteen-week-old *TDAG51*^*−/−*^ mice injected with AAV-GFP or AAV-TDAG51-GFP fed HFD were fasted for 6 h and livers collected 15 min after injection of insulin. Representative immunoblots for total and phospho-Akt (S473) from liver lysates. N = 4 mice per group. Data represent the mean ± SD (∗∗*p* = 0.01). Statistical comparisons were assessed by an independent Student’s t tests (two-tailed). AAV, adeno-associated virus; HFD, high-fat diet; TDAG51, T-cell death–associated gene 51.

At 8 weeks postinjection (4 weeks on HFD), restoring hepatic TDAG51 expression in *TDAG51*^*−/−*^ mice did not significantly improve response to a glucose challenge (Fig. 4*A*). In contrast, a significant improvement in response to an insulin challenge was observed in *TDAG51*^*−/−*^ mice receiving the TDAG51-GFP AAV compared to mice receiving the GFP AAV (Fig. 4*B*). Restoration of TDAG51 in the livers of *TDAG51*^*−/−*^ mice injected with insulin also significantly improved insulin sensitivity as measured by downstream Akt phosphorylation at Ser473 (Fig. 4*C*).

**Figure 5:**
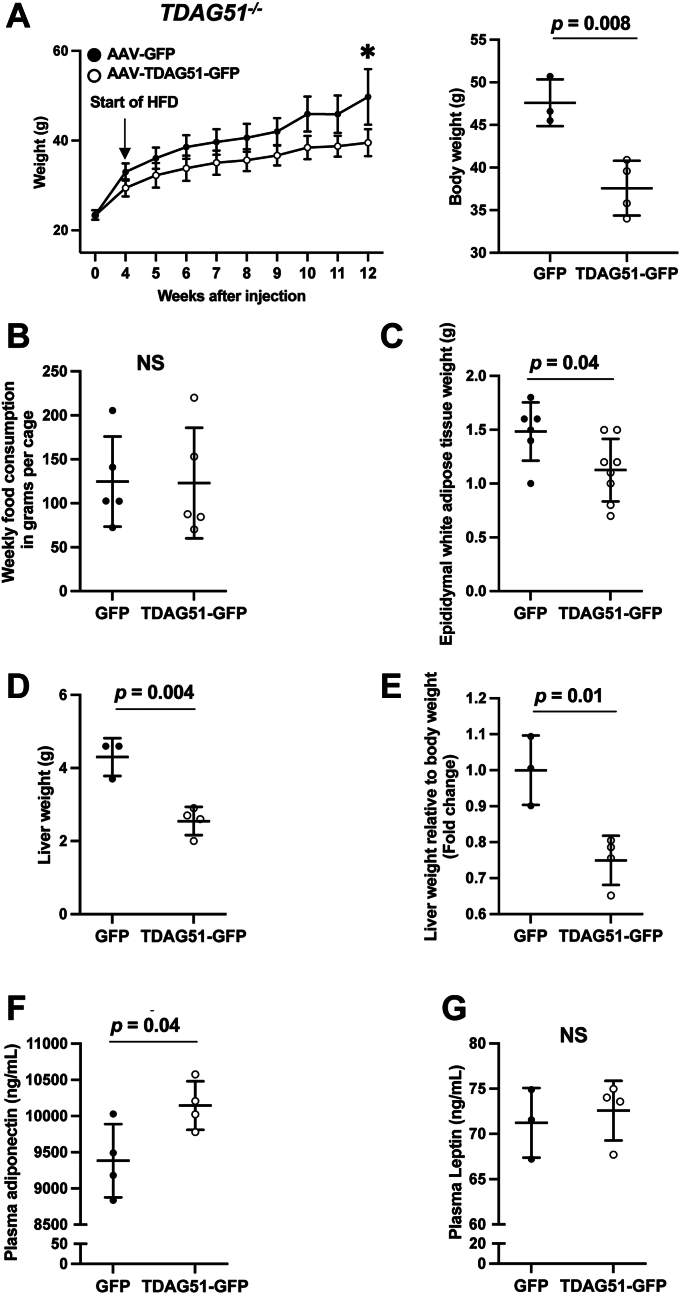
**Hepatic TDAG51 AAV reduces body weight,in addition toliverand adipose tissue weight and results in higher serum adiponectin levels in *TDAG51***^***−/−***^**mice fed HFD.***A*, total body weight of AAV-injected *TDAG51*^*−/−*^ mice fed HFD over time and at endpoint. Groups were compared by two-way ANOVA using repeated measures (Time × AAV: *F* = 5.99, *p* < 0.0001; time: *F* = 138.6, *p* < 0.0001; AAV: *F* = 0.06, *p* = 0.057). Sidak’s multiple comparison used post hoc (12 weeks, *p* < 0.002). Data represent the mean ± SD. At endpoint, groups were compared with an independent Student’s *t* tests (two-tailed) (∗∗*p* = 0.008). *B*, food intake of AAV-injected *TDAG51*^*−/−*^ HFD mice. *C*, epididymal fat pad weight at endpoint from AAV-injected *TDAG51*^*−/−*^ mice fed HFD (∗*p* = 0.04). *D*, total liver weight at endpoint from AAV-injected *TDAG51*^*−/−*^ mice fed HFD (∗∗*p* = 0.004). *E*, liver weight as a percentage of total body weight in *TDAG51*^*−/−*^ mice fed HFD (∗∗*p* = 0.01). *F*, plasma adiponectin levels at endpoint from AAV-injected *TDAG51*^*−/−*^ mice after an overnight fast (∗*p* = 0.04). *G*, plasma leptin levels at endpoint from AAV-injected *TDAG51*^*−/−*^ mice after an overnight fast. N = 3 to 4 mice per group. Data represent the mean ± SD. *B*–*G*, statistical comparison of two groups were compared with independent Student’s *t* tests (two-tailed). AAV, adeno-associated virus; HFD, high-fat diet; TDAG51, T-cell death–associated gene 51.

To assess the effects of ectopic hepatic TDAG51 protein expression on body weight, *TDAG51*^*−/−*^ mice were weighed weekly following the initial injection with AAV-GFP or AAV-TDAG51-GFP (Fig. 5*A*). After 4 weeks postinjection and prior to HFD feeding, mice receiving the AAV-TDAG51-GFP weighted approximately 3.5 g less than mice receiving the AAV-GFP (29.5 g *versus* 33.0 g, respectively; *p* < 0.05, n = 4–5). Restoring TDAG51 in *TDAG51*^*−/−*^ mice significantly reduced total body weight at the study endpoint after HFD feeding (Fig. 5*A*), despite no significant differences observed in food consumption between the two groups (Fig. 5*B*).

The reduction in weight gain for AAV-TDAG51-GFP–injected mice can be attributed in part to the reduction in epididymal adipose tissue (Fig. 5*C*) and liver (Fig. 5, *D* and *E*) weight. Furthermore, restoring hepatic TDAG51 protein level in *TDAG51*^*−/−*^ mice resulted in significantly higher circulating levels of adiponectin (Fig. 5*F*) without altering leptin levels (Fig. 5*G*).

**Figure 6:**
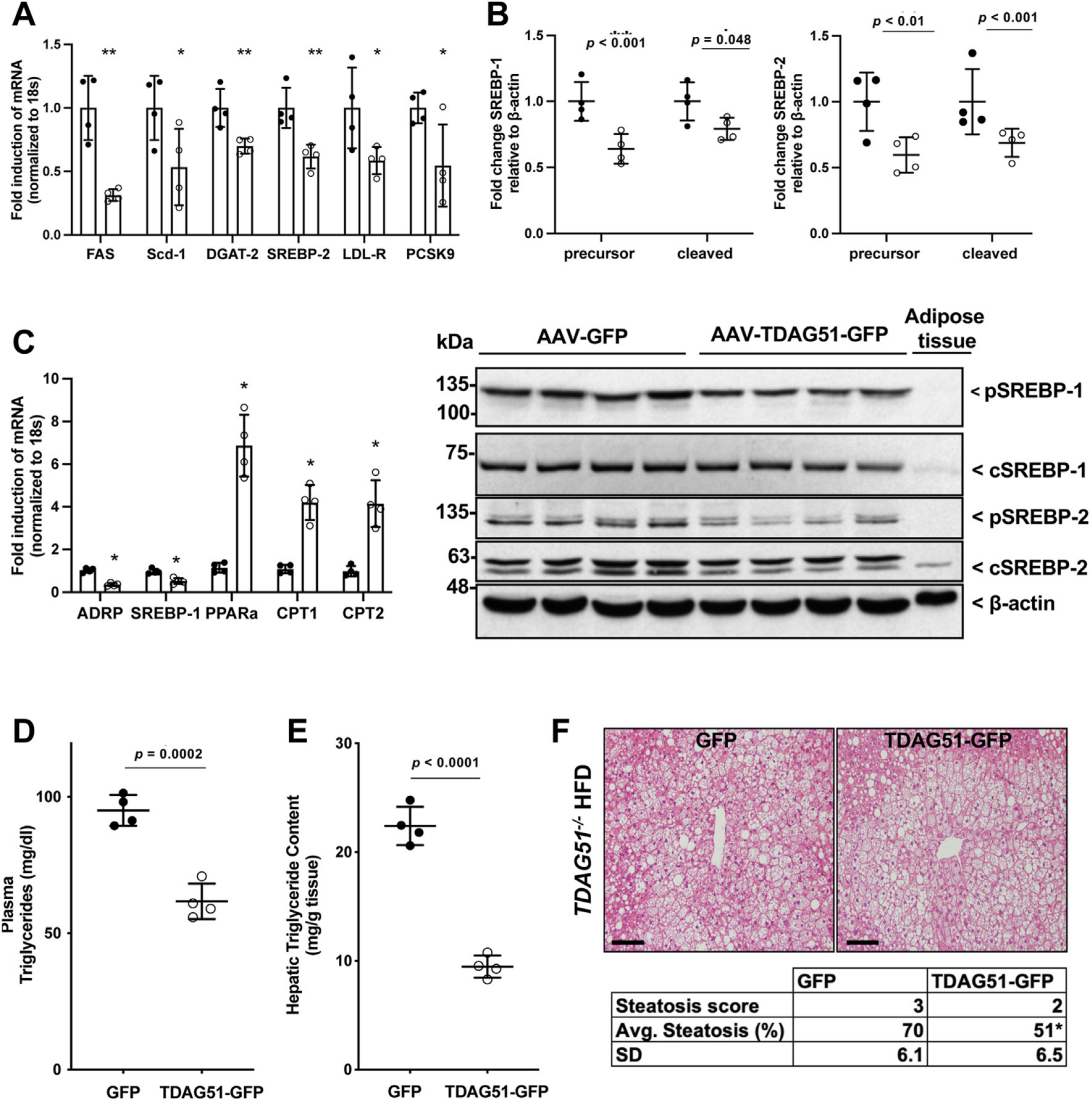
**Restoration of hepatic TDAG51 protein reduces total hepatic lipid content by increasing hepatic gene expression ofbeta-oxidation markers while reducing *de novo* lipogenesis.***A*, twenty-six-week-old *TDAG51*^*−/−*^ mice injected with AAV-GFP or AAV-TDAG51-GFP fed HFD were fasted for 16 h and liver RNA and total protein lysates were collected and subjected to RT-PCR and immunoblotting. Fold change in *FAS*, *Scd-1, DGAT-2, SREBP-2, LDLR,* and *PCSK9* mRNA expression in AAV-injected *TDAG51*^*−/−*^ mice fed HFD. *B*, representative immunoblots for precursor (p) and cleaved (n) SREBP1 and SREBP2 from liver lysates. N = 4 mice per group. *C*, Fold change in *ADRP*, *SREBP-1*, *PPAR-alph*a, *CPT1*, and *CPT2* normalized to 18S housekeeping gene and relative to AAV-GFP controls. *D*, plasma (mg/dl) and *E*, hepatic (mg/g tissue) triglyceride content measured by a colorimetric assay. *F*, representative images of H&E-stained livers from *TDAG51*^*−/−*^ mice (*bottom panels*). The scale bar represents 10 μm. Table represents pathologist scoring of percent steatosis per 20× liver tissue. Statistical comparison of two groups were compared with independent Student’s *t* tests (two-tailed), ∗∗*p* < 0.01 *versus* GFP and ∗*p* < 0.05 *versus* GFP. *A*–*E*, data represent the mean ± SD. AAV, adeno-associated virus; DGAT-2, diacylglycerol *O*-acyltransferase-2; FAS, fatty acid synthase; HFD, high-fat diet; Scd-1, stearoyl-CoA desaturase-1; SREBP, sterol regulatory–element binding protein; TDAG51, T-cell death–associated gene 51.

Increasing hepatic TDAG51 levels in *TDAG51*^*−/−*^ mice caused a significant reduction in the expression of sterol regulatory–element binding protein (*SREBP*)-1c and several target genes measured in liver tissue, including fatty acid synthase (*FAS*) and stearoyl-CoA desaturase-1 (*Scd-1*) (*p* < 0.05) (Fig. 6*A*). Furthermore, increasing hepatic TDAG51 protein levels significantly reduced the expression of *diacylglycerol* *O-acyltransferase*, the enzyme that catalyzes the final step in the production of triglycerides (22) (Fig. 6*A*). Increasing hepatic TDAG51 expression in *TDAG51*^*−/−*^ mice similarly caused a significant reduction in *SREBP2* transcript and its downstream targets *LDLR* and *PCSK9* (Fig. 6*A*). Consistent with reductions in *SREBP1/2* transcript, SREBP-1 and SREBP-2 precursor and cleaved proteins were significantly reduced in livers harvested from AAV-TDAG51-GFP–injected mice (Fig. 6*B*). Along with a significant reduction in SREBP-1 transcript, several fatty acid oxidation genes were also induced in AAV-TDAG51-GFP–injected mice compared to GFP controls (Fig. 6*C*). Furthermore, the reduction of these drivers of triglyceride synthesis produced a significant reduction in plasma and hepatic triglyceride content in AAV-TDAG51-GFP–injected mice compared to GFP controls (Fig. 6, *D* and *E*). Representative H&E-stained images scored by a pathologist blinded to the treatment groups indicated a significant reduction in percent steatosis in *TDAG51*^*−/−*^ mice fed HFD expressing hepatic TDAG51-GFP compared to GFP (Fig. 6*F*).

### Overexpression of hepatic TDAG51 protein reduces total lipid content in cultured hepatocytes by decreasing cleaved SREBP-1 protein expression

**Figure 7:**
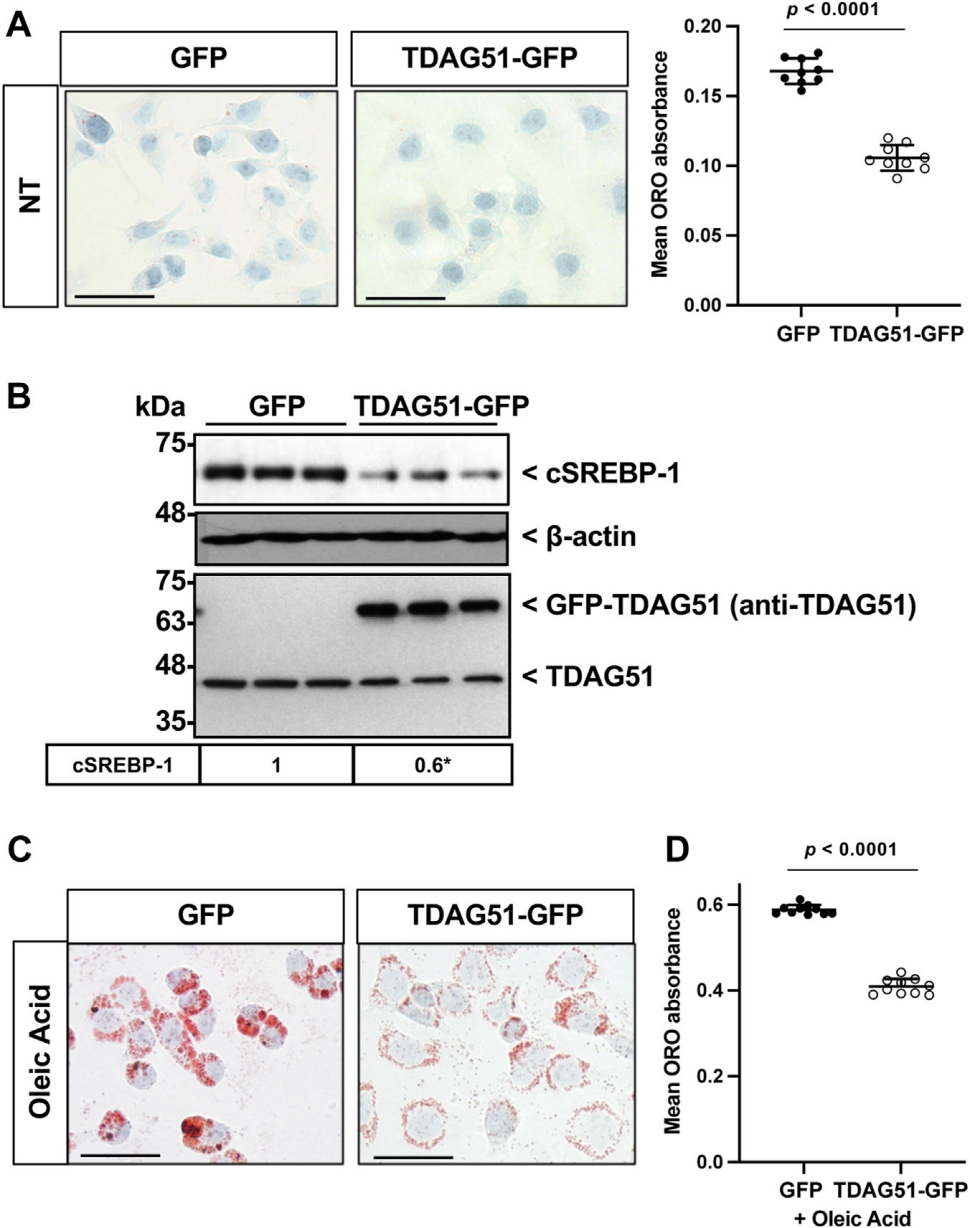
**Overexpression of hepatic TDAG51 protein reduces total hepatic lipid content *in vitro* by decreasing cleaved SREBP-1 protein expression.***A*, Huh7 cells transfected with GFP or TDAG51-GFP plasmid then stained and quantified for Oil Red O. *B*, representative immunoblots for SREBP-1, GFP, and GFP-TDAG51 from Huh7 cells transfected with GFP or GFP-TDAG51. *C*, Huh7 cells transfected with GFP or TDAG51-GFP plasmid were treated with oleic acid then stained and quantified for Oil Red O. *D*, total triglyceride content measured in Huh7 cell lysate transfected with GFP or GFP-TDAG51 and treated with oleic acid. Data represent the mean ± SD. Statistical comparison of two groups were compared with independent Student’s *t* tests (two-tailed). ∗*p* < 0.05 *versus* GFP. The scale bar represents 100 μm. AAV, adeno-associated virus; SREBP, sterol regulatory–element binding protein; TDAG51, T-cell death–associated gene 51.

The lipid lowering effects of TDAG51 overexpression was examined in human hepatocytes. Huh7 cells transfected with GFP or GFP-TDAG51 expression plasmid were treated with oleic acid for 24 h, then quantified for Oil Red O (ORO) staining relative to protein content (Fig. 7, *A* and *C*). Overexpression of TDAG51 protein resulted in a marked reduction in ORO stain, a strong indicator of lipid content. To further examine TDAG51’s ability to affect hepatocyte lipid content, cleaved SREBP-1 was measured. Overexpression of TDAG51 caused a significant reduction in cleaved SREBP-1, a finding consistent with our *in vivo* study (Fig. 7*B*).

### Restoring TDAG51 expression in ob/ob mice increases hepatic response to insulin and reduces total body weight

**Figure 8:**
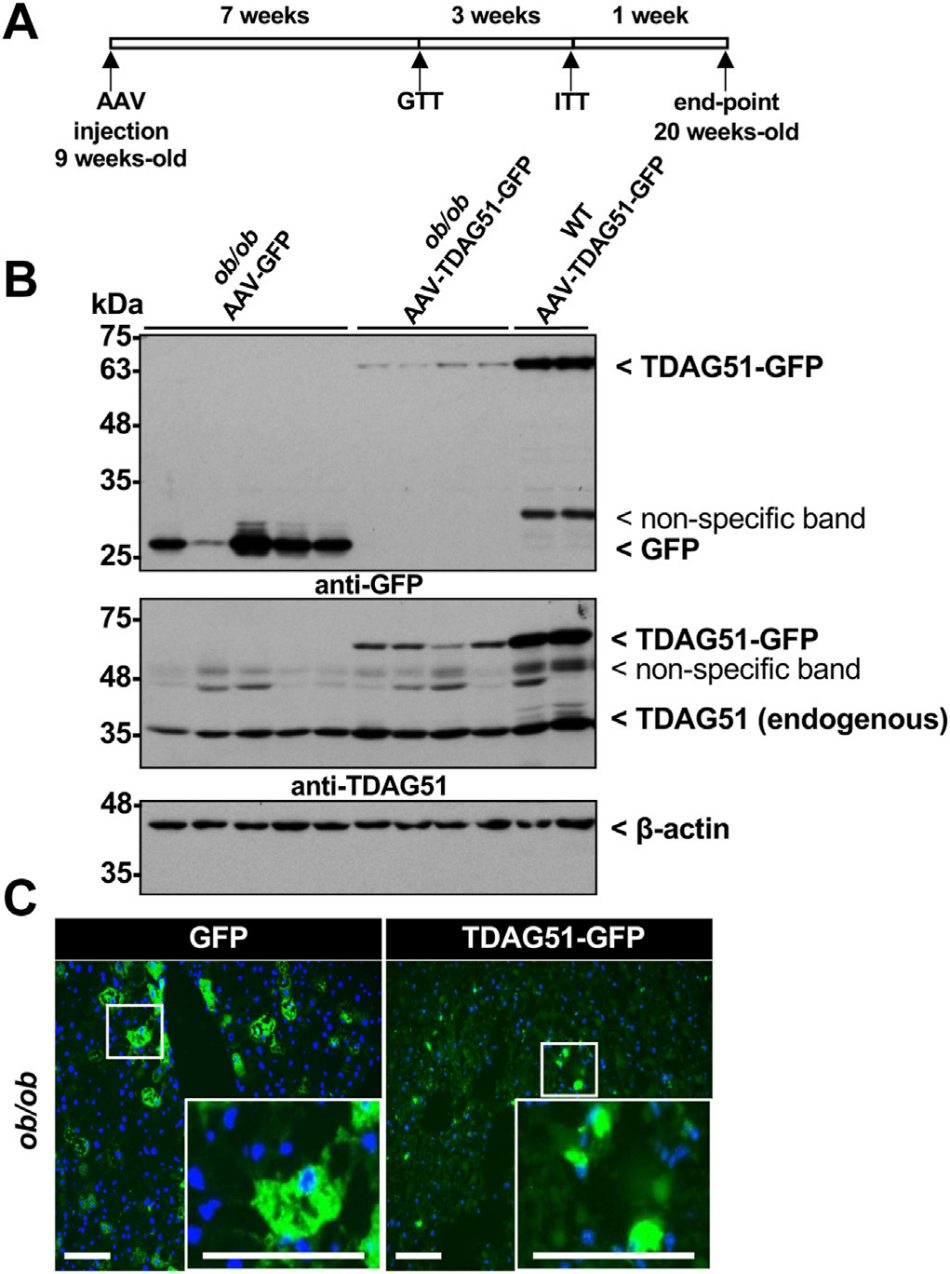
**Expression of hepatic TDAG51 AAV in *ob/ob* mice.***A*, nine-week-old *ob/ob* mice were injected with 5 × 10^11^ genome containing particles of AAV-GFP or AAV-TDAG51-GFP. At 16 and 19 weeks of age, glucose tolerance (GTT) and insulin tolerance tests (ITT) were performed, respectively. At 20 weeks of age (11 weeks post-injection), *ob/ob* mice were sacrificed and livers harvested. Total liver lysates immunoblotted for GFP, TDAG51, or TDAG51-GFP. Immunoblots were reprobed for β-actin as a loading control. *B*, representative immunoblots from livers of *ob/ob* mice injected with AAV-GFP or AAV-TDAG51-GFP. Densitometric analysis revealed that ectopic TDAG51-GFP protein levels were significantly reduced in chow-fed *ob/ob* mice compared to WT mice injected with AAV-TDAG51-GFP. *C*, GFP fluorescent images of liver sections from AAV-GFP– or AAV-TDAG51-GFP–infected *ob/ob* mice. *Green* represents GFP while *blue* represents 4′,6-diamidino-2-phenylindole (nuclei). The scale bar represents 10 μm. AAV, adeno-associated virus; TDAG51, T-cell death–associated gene 51.

The leptin-deficient (*ob/ob*) mouse model was previously shown to exhibit significantly diminished hepatic TDAG51 protein expression (10). Figure 8*A* outlines the experimental timeline for 9-week-old chow-fed *ob/ob* mice injected with either AAV-GFP or AAV-TDAG51-GFP. GFP and TDAG51-GFP protein were detectable in the livers of *ob/ob* mice injected with AAV-GFP and AAV-TDAG51-GFP, respectively (Fig. 8*B*). Consistent with our findings in *TDAG51*^*−/−*^ mice, ectopic expression of hepatic TDAG51-GFP fusion protein was significantly reduced by approximately 88% (*p* < 0.01) in *ob/ob* mice compared to TDAG51-GFP protein expression in age- and duration-matched WT chow-fed mice (Fig. 8*B*). Consistent with the immunoblot analysis, the number and intensity of GFP-positive hepatocytes were markedly increased in *ob/ob* mice treated with AAV-GFP compared to the AAV-TDAG51-GFP–injected mice (Fig. 8*C*). The marked decrease in hepatic TDAG51-GFP is evident in NAFLD mouse models independent of a HFD or leptin signaling.

**Figure 9:**
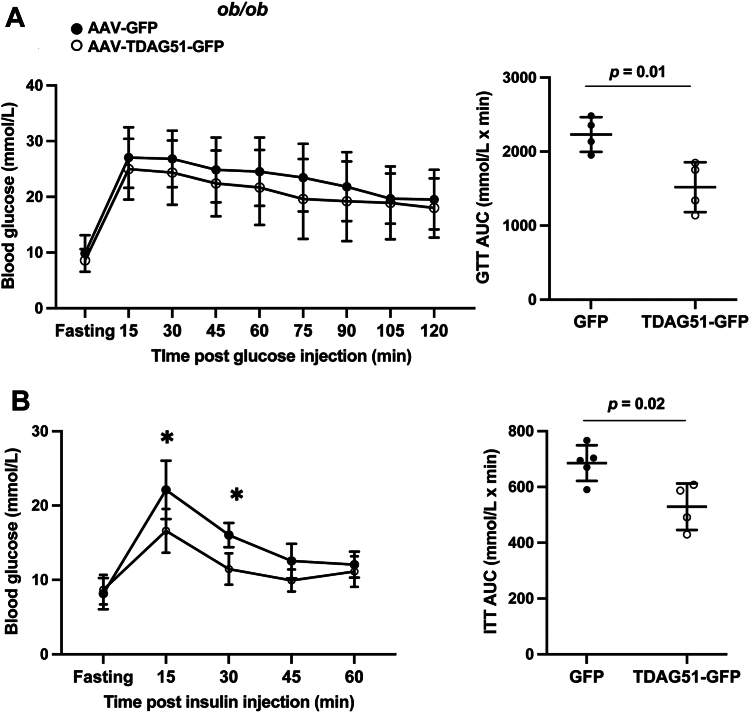
**Restoration of hepatic TDAG51 protein improves insulin sensitivity in the livers of *ob/ob* mice.***A*, glucose tolerance test (GTT) following 6 h fast of *ob/ob* mice, 10 weeks post-injection with AAV-GFP or AAV-TDAG51-GFP (*left panel*). Groups were compared by two-way ANOVA using repeated measures (Time × AAV: *F* = 0.47, *p* > 0.05; time: *F* = 30.4, *p* < 0.0005; AAV: *F* = 0.33, *p* < 0.02). Data represent the mean ± SD. Area under the curve (AUC) is represented in the *right panel*, groups were compared by student’s independent *t* test (two-tailed) (*p* = 0.01). Data represent the mean ± SD. *B*, insulin tolerance test (ITT) of *ob/ob* mice, 10 weeks post-injection with AAV-GFP or AAV-TDAG51-GFP (*left panel*). Groups were compared by two-way ANOVA using repeated measures (Time × AAV: *F* = 3.58, *p* < 0.02; time: *F* = 38.33, *p* < 0.0001; AAV: *F* = 6.26, *p* < 0.05). Sidak’s multiple comparison used post hoc (15 min, ∗∗*p* < 0.01; 30 min, ∗*p* < 0.04). Data represent the mean ± SD. AUC is represented in the *right panel*; groups were compared by student’s independent *t* test (two-tailed) (*p* = 0.02). Data represent the mean ± SD. AAV, adeno-associated virus; TDAG51, T-cell death–associated gene 51.

At 8 weeks post-injection, GTTs indicated that restoring TDAG51 protein expression in *ob/ob* mice significantly increases the response to a glucose challenge (Fig. 9*A*). In addition, restoring TDAG51 protein levels in the *ob/ob* mice significantly increases response to an insulin challenge as indicated by a significant decrease in the area under the curve (Fig. 9*B*).

**Figure 10:**
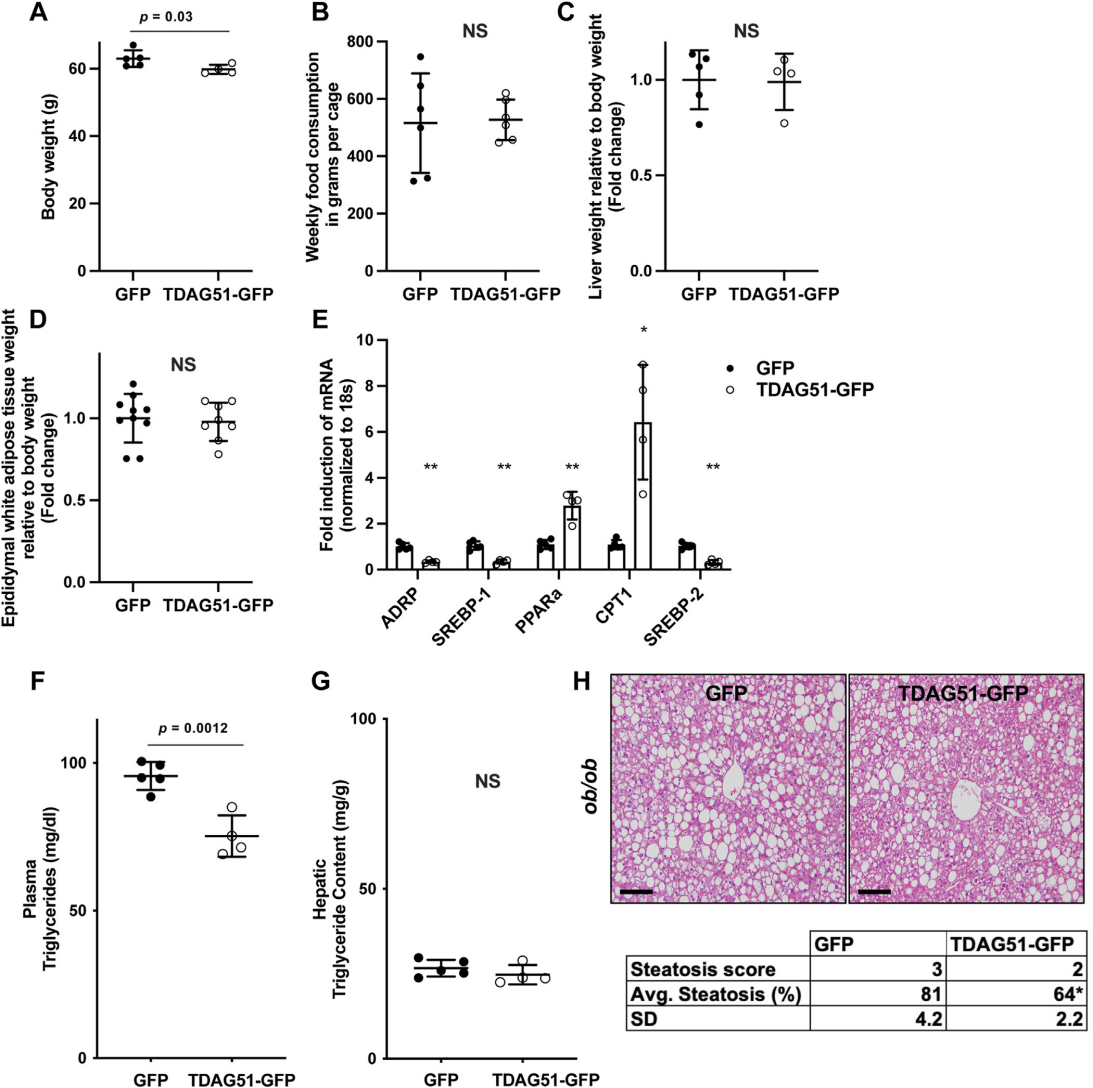
**Restoration of hepatic TDAG51 protein reduces body weight and plasma triglycerides by increasing gene expression ofbeta-oxidation markers while reducing *de novo* lipogenesis in the livers of *ob/ob*.***A*, total body weight at endpoint (*p* = 0.03). *B*, food intake of AAV-injected *ob/ob* mice. *C*, total raw liver weight expressed as a percent of body weight. *D*, epididymal fat pad weight expressed as a percent of body weight. *E*, fold change of lipogenic and beta-oxidative markers normalized to 18S relative to AAV-GFP control. *F*, plasma (mg/dl) (*p* = 0.0012) and (*G*) hepatic (mg/g tissue) triglyceride content measured by a colorimetric assay. *H*, representative images of H&E-stained livers from *ob/ob* mice injected with AAV-GFP or AAV-TDAG51-GFP scored for percent steatosis. The scale bar represents 10 μm. *A*–*H*, data represent the mean ± SD. Statistical comparison of two groups were compared with independent Student’s *t* tests (two-tailed). ∗*p* < 0.05 and ∗∗*p* < 0.01. AAV, adeno-associated virus; TDAG51, T-cell death–associated gene 51.

Restoring hepatic TDAG51 protein in *ob/ob* mice significantly reduced weight gain at study endpoint (Fig. 10*A*) with no changes in food consumption (Fig. 10*B*). At endpoint, liver and epididymal white adipose tissue normalized to body weight were not significantly reduced in AAV-TDAG51-GFP–injected mice (Fig. 10, *C* and *D*). However, *SREBP-1/2* were significantly reduced at the transcript level while oxidation markers were significantly increased (Fig. 10*E*). Consistent with these findings, plasma triglyceride content was significantly reduced but hepatic triglyceride content was not (Fig. 10, *F* and *G*). Representative H&E-stained images scored by a pathologist blinded to the treatment groups indicated a significant reduction in percent steatosis in *ob/ob* mice fed HFD expressing hepatic TDAG51-GFP compared to GFP (Fig. 10*H*).

## Discussion

Our study provides evidence that the marked loss of hepatic TDAG51 is evident in multiple mouse models of liver steatosis and injury. In support of these observations in mouse liver tissue, we demonstrate a similar reduction in hepatic PHLDA1 protein, the human homolog of TDAG51, in NASH patients. These findings suggest that TDAG51/PHLDA1 is an integral marker and regulator of liver health. Identifying the mechanisms causing hepatic TDAG51 degradation highlights key events that result in NAFLD and obesity. Additional studies have demonstrated that liver proteins are targeted for degradation following liver injury/steatosis, including nerve growth factor (23) as well as the CASP8 and FADD-like apoptosis regulator (24). Importantly, restoration of these proteins is protective in the context of cardiomyopathy and NASH, respectively (23, 24).

The forced ectopic expression of TDAG51-GFP in the *TDAG51*^*−/−*^ and *ob/ob* mice is consistently lower than TDAG51-GFP expression in the age-matched WT chow-fed controls. The protective effect of restoring hepatic TDAG51 protein level appears to be directly relevant to the stability of the TDAG51 fusion protein under conditions of liver injury. This observed reduction in TDAG51 fusion protein is not a general phenomenon of hepatic protein expression using AAV, since the GFP protein expression does not change dramatically across groups.

Histological and biochemical examination of livers isolated from *TDAG51*^−/−^ mice with AAV-TDAG51-GFP restoration after insulin injection revealed a remarkable contrast to those from GFP controls. We demonstrate that AAV-TDAG51-GFP clearly increases the downstream insulin signaling marker, p-Akt, in *TDAG51*^−/−^ mice upon insulin injection, suggesting an improvement in insulin sensitivity. Although chow-fed *TDAG51*^−/−^ exhibit higher fasting blood glucose and insulin levels (10), increasing TDAG51 protein levels enhances insulin sensitivity in HFD-fed mice subjected to an insulin tolerance test (ITT). This study demonstrates that TDAG51-GFP restoration improved insulin sensitivity, while no changes were observed after a glucose challenge in *TDAG51*^−/−^ mice, confirming the originally reported basal dysglycemia and hyperglycemia observed in these mice (10). However, at endpoint, plasma and hepatic triglyceride content were significantly reduced in TDAG51-GFP mice compared to GFP controls, consistent with the reductions in liver and total body weight. Interestingly, increasing hepatic TDAG51 protein in *TDAG51*^*−/−*^ mice reduced total body weight prior to the start of high-fat feeding, while no changes in food consumption were observed throughout the study. To support these findings, we compared fasting plasma leptin, a key regulator of food intake suppression (25, 26), between AAV-injected animals and found no significant differences. Since circulating adiponectin levels are inversely related to obesity and type 2 diabetes (27), we also assayed fasting plasma adiponectin in the *TDAG51*^*−/−*^ mouse cohort. Increasing hepatic TDAG51 protein levels in *TDAG51*^*−/−*^ mice resulted in significantly higher levels of plasma adiponectin. This is consistent with the previous finding that *TDAG51*^*−/−*^ mice exhibit significantly lower levels of adiponectin than WT mice (10). Although there is no evidence that the TDAG51-GFP fusion protein is secreted, TDAG51 may exert regulatory crosstalk between liver and adipose tissue based on the changes in adiponectin secretion. The mechanism by which TDAG51 exhibits remote effects in tissues where it is not exogenously expressed is unknown and will be the subject of future investigation.

The benefits of restoring TDAG51 as a means of inhibiting the expression of the SREBPs and their downstream target was investigated based on our prior observations where *TDAG51*^*−/−*^ mice have increased hepatic activation of SREBP-1 compared to WT littermate controls (10). SREBP-1c is the predominant form found in the liver and adipose, with an established role in regulating fatty acid synthesis and lipogenesis (28, 29, 30, 31). Increasing hepatic TDAG51 protein level in TDAG51^−/−^ mice reduces SREBP-1c activation as measured by *SREBP-1c* transcript and it's target genes *Scd-1* and *FAS*. These findings of TDAG51 overexpression resulting in the reduction in SREBP-1 are consistent with a recent report showing that TDAG51 overexpression can inhibit the SREBP-1/angiopoietin-like 8 pathway which led to significant improvements in glucose, insulin, and lipid metabolism in a mouse model of gestational diabetes mellitus (32). The reduction in body and liver weight as a result of reduced SREBP activation is further supported by reports of FAS inhibitors showing a similar effect on weight loss (33). Consistent with this concept, restoration of TDAG51 in *TDAG51*^−/−^ mice resulted in a significant reduction of SREBP-2 expression and downstream factors, along with an increase in β-oxidation genes compared to GFP controls. The relationship between TDAG51 and its ability to reduce SREBP-1 expression was further examined in the human liver carcinoma Huh7 cell line. Overexpression of TDAG51 significantly reduced cleaved SREBP-1 protein and reduced hepatic lipid uptake of hepatocytes treated with oleic acid. This data is in accordance with previous reports that the overexpression of SREBP-1 induced lipogenic enzymes and resulted in fatty livers in several mouse models, including SREBP-1a and -1c transgenic mice (34, 35, 36). Attenuation of SREBP-1 has been shown to reduce steatosis and protect against NAFLD (28, 37, 38, 39, 40).

Leptin-deficient mice develop obesity, and fatty livers due to inherent deficiency of the appetite-suppressing hormone, leptin (41, 42, 43, 44, 45). These genetically modified mice present the most severe obesity reported in rodents (46). Increasing levels of TDAG51 in *ob/ob* mice had a significant sensitizing effect on insulin and glucose tolerance compared to GFP controls. Upon AAV-TDAG51-GFP overexpression, *ob/ob* mice exhibit reduced body weight, in addition to significant reductions in plasma triglyceride content. Although overexpression of TDAG51 significantly reduced *SREBP-1c* transcript, this was insufficient to cause a reduction in hepatic triglycerides, possibly due to the markedly higher levels of plasma lipid in these mice.

Degradation of hepatic TDAG51 protein under conditions of NAFLD can potentially be attributed to autophagy-mediated clearance of proteins. Autophagy is a tightly regulated, stress-induced lysosomal degradative pathway that is highly selective for macromolecules and organelles (47, 48). Previous studies have reported that autophagy directly impacts insulin tolerance, hepatic triglyceride levels, and obesity (49, 50, 51). TDAG51 gene expression is reportedly involved in the maturation of autophagosomes (52). Furthermore, our group has previously demonstrated that TDAG51 protein expression colocalizes with large perinuclear vesicle structures (peroxisomes) and small vesicle structures (lysosomes), increasing the likelihood that TDAG51 facilitates autophagy and results in the turnover of this protein (53, 54). Consistent with these findings, we have demonstrated a robust impairment of autophagy in the livers of *TDAG51*^*−/−*^ mice (Yousof and Austin, unpublished results). Thus, it is possible that TDAG51 plays an important role through autophagic induction, which can potentially lead to its degradation under conditions of NAFLD-related dysregulated autophagy.

In summary, TDAG51 protein expression is dramatically reduced in multiple mouse models of NAFLD and liver injury as well as in human NASH liver samples compared to healthy controls. Results from this study indicate AAV-TDAG51-GFP protein levels were significantly lower than AAV-GFP in WT controls. Despite this, even partial rescue of TDAG51 elicits significant beneficial effects in both genetic- and diet-induced models of obesity which endogenously express significantly low or undetectable levels of hepatic TDAG51 protein. Partial restoration of AAV-TDAG51 reduced weight gain, increased insulin sensitivity, and caused a significant reduction in hepatic expression of a series of lipogenic genes, increased beta-oxidation markers and reduced plasma triglyceride levels in *TDAG51*^−/−^ and *ob/ob* mice, indicating that TDAG51 may regulate triglyceride accumulation in circulation by mediating the expression of lipogenic enzymes. Overexpression of PHLDA1 protein in human hepatocytes negatively regulates SREBP-1 protein, a crucial regulator of triglyceride synthesis.

It can be concluded, therefore, that partial restoration of TDAG51 regulates hepatic gene expression to modulate lipid biosynthesis and deposition in the liver. Thus, future investigations aimed at partially restoring or moderately increasing hepatic TDAG51 protein levels and/or stability may be just as beneficial at reducing IR and weight gain.

## Experimental procedures

### Adeno-associated virus

Customized adeno-associated viral vectors encoding TDAG51 were created by Vector BioLabs. Constructs consisted of a capsid from serotype 8 and inverted terminal repeat from serotype 2 (55). The TDAG51-GFP expression vector contained an albumin promoter with hF9 enhancer and 3′ intron adjacent to the mouse *TDAG51* gene with the *GFP* gene inserted at the C terminus that allows for hepatic overexpression of a TDAG51-GFP fusion protein. Similar TDAG51-GFP constructs have previously been developed to investigate the role of TDAG51 in apoptosis (54), suggesting that GFP does not interfere with TDAG51 function (54, 56, 57, 58, 59). The control virus encoded only *GFP* in place of the *TDAG51-GFP* complimentary DNA (cDNA) insert. Mice were injected with 5 × 10^11^ genome containing (GC/ml) particles *via* tail vein under isoflurane anesthetic, as per the AAV manufacturer’s recommendation.

### Animals and diets

Several independent mouse models of hepatic steatosis and/or injury were utilized in this study. The first model utilized 14-week-old male C57Bl/6 mice fed a methionine-deficient diet (DYET#518810 Dyets Inc) for 18 days compared to control mice receiving standard chow (LabDietNIH5K67, PMI nutrition international) (n = 8). Heterozygous *CBS*^*±*^ breeding pairs were used to generate *CBS*^*−/−*^ mice designated as “MKOs” (The Jackson Laboratory). Mice were crossbred to JAX C57BL/6J mice for seven generations. Fourteen-week-old male human transgenic (HO) mouse model has been described previously (16). Animals were individually housed and maintained on standard chow (PMI nutrition international). MKO exhibit severe liver injury with microsteatosis and extensive fibrosis, while HO mice exhibit very mild liver injury with no steatosis or fibrosis detectable and only 2-fold increase in plasma alanine transaminase levels (15, 16). Two 350 μg/g APAP doses were supplemented to male HO and WT mice 4 h apart. Corresponding nontreated male HO and WT controls were incorporated into the study and all groups were sacrificed 6 h after the second injection (n = 8). Male WT C57Bl/6 mice were injected *via* tail vein with 5 × 10^11^ GC/ml of AAV at 9 weeks of age (The Jackson Laboratory). Mice fed a standard chow diet (4% of kcal from fat, Harlan Teklad) were sacrificed at 12 weeks of age or 20 weeks of age to confirm viral expression. *TDAG51*^*−/−*^ mice were previously generated (60) and back-crossed onto a C57Bl/6 background for at least nine generations and genotyped. Male mice were injected with the AAV at 15 weeks of age (n = 4). Four weeks after 5 × 10^11^ GC/ml of AAV was injected *via* tail vein, the mice were placed on a HFD (Harlan Teklad). Glucose and ITTs were performed at 8 weeks post-injection. The mice were sacrificed at 27 weeks of age (12 weeks post-injection). Figure 3*A* outlines the experimental timeline for these *in vivo* studies. The male *ob/ob* mice on a C57Bl/6 background were purchased from The Jackson Laboratory, and injected *via* tail vein with 5 × 10^11^ GC/ml of AAV at 9 weeks of age (n = 5) and fed a chow diet for the duration of the experiments (Harlan Teklad). Glucose and insulin tolerance testing was performed at 10 weeks postinjection. The mice were sacrificed at 20 weeks of age (11 weeks post-injection). Figure 5*A* outlines the experimental timeline for the *ob/ob* mouse studies. Following injection with AAV, mice were housed in an Ultraclean Unit and handled under a laminar flow biosafety cabinet. Mice not injected with the AAV were housed in the Specific Pathogen–Free Unit. Mice were maintained within the St Joseph’s Healthcare Animal Facility under 12-h light-dark cycles, *ad libitum*. Food and water intake were monitored biweekly. Mice were sacrificed under isoflurane anesthetic after perfusing the circulation with PBS. A portion of the tissues were immediately snap-frozen in liquid nitrogen until long-term storage at −80 °C, stored in formalin until histological processing, or were placed in optical cutting temperature gel (VWR) and immediately frozen over dry ice until long-term storage at −20 °C. All protocols for animal use and euthanasia were approved by the McMaster University Animal Research Ethics Board.

### Human liver samples

Post-mortem liver tissue and liver biopsies were acquired and scored for diagnosis by an NIH pathologist using the NAFLD activity scoring system (19). Whole-cell liver lysates were generated for Western blot analyses from individual liver tissue samples (n = 7) as previously described (19). NASH fatty (n = 5) and NASH nonfatty (n = 2) data were combined in the analysis into one category designated as NASH due to the lack of global gene expression changes and mechanistic differences between the two categories as reported previously (61). Total protein content was analyzed using the Pierce BCA (Thermo Fisher Scientific) protein quantitation assay.

### Western blotting and antibodies

Mouse tissues were homogenized with radioimmunoprecipitation assay buffer plus complete protease inhibitor cocktail (Roche). Lysates were centrifuged at 16,000*g* for 20 min at 4 °C. Protein concentrations were determined by Lowry protein assay (Bio-Rad). Samples were prepared in 6× Laemmli buffer prior to boiling the samples for 5 min at 100°C, whereby 40 μg of protein was resolved on 10% SDS-polyacrylamide gels under denaturing conditions. Following separation, gels were then transferred onto a nitrocellulose membrane (Bio-Rad), as previously described (10). Human liver lysates were separated on 7.5% SDS polyacrylamide gels, and then transferred onto polyvinylidene fluoride membranes, and immunoblotted as previously described (62). The following antibodies were used for immunoblotting: GFP (Aves), SREBP-1 (Santa Cruz Biotechnology), SREBP-2 (Abcam), TDAG51 (Santa Cruz Biotechnology), pS473 Akt (Cell Signaling Technology), total Akt (Cell Signaling Technology), β-actin (Sigma-Aldrich), GAPDH (Cell Signaling), extracellular signal-regulated kinase 2 (Santa Cruz, California), MRP2 (Sigma-Aldrich), and MRP4 (Abcam). The specificity of these antibodies were validated as previously described (10, 19, 61, 62). Following primary antibody incubation, membranes were incubated with horseradish peroxidase–conjugated secondary antibodies diluted in 1 × tris-buffered saline with 1% milk and 0.1% Tween 20. Membranes were visualized using an enhanced chemiluminescent reagent (FroggaBio) on Amersham Biosciences Hyperfilm (GE Healthcare), which was subsequently developed on a Kodak X-Omat 1000A processor. Following exposure, band intensities were quantified with ChemiDoc XRS+ Molecular Imager (Bio-Rad) and analyzed with ImageLab software (BioRad: https://www.bio-rad.com/en-ca/product/image-lab-software?ID=KRE6P5E8Z).

### Histology

Mouse tissue was excised and placed in formalin for 18 h. After a series of alcohol dehydrations (St Joseph’s Healthcare Hamilton Histology Department), tissues were embedded in paraffin blocks and 4 μm thick sections were used for H&E staining. Stained H&E liver sections were imaged at 20×. A pathologist blindly scored 5 images per animal (n = 4–5) using the NAFLD scoring system by the NASH Clinical Research Network (19, 63). Steatosis was calculated as a percentage of lipid droplets per 20× field. Analysis of intracellular triglyceride accumulation was performed in cultured cells fixed in 4% paraformaldehyde using ORO. The ORO content of isopropanol extracts was measured using a spectrophotometer (Molecular Devices) at a wavelength of 520 nm (64).

### Biochemical analyses

Plasma adiponectin (R&D Systems) and leptin (Linco) concentrations were measured using mouse ELISA kits. Liver tissue and serum was assessed for triglyceride content using a triglyceride assay kit (Abcam) according to manufacturer’s instructions. All data were normalized to tissue weight.

### Metabolic studies

For GTTs, mice were fasted for 16 h and given an intraperitoneal injection of glucose (1.5 mg/g body weight). For ITTs, mice were fasted for 6 h and injected intraperitoneally with 1 unit of insulin per kg for *TDAG51*^−^*^/^*^−^ mice and 1.5 units of insulin per kg for *ob/ob* mice. Blood was collected from the tail vein, and glucose concentrations were measured using a hand-held glucometer. For *in vivo* insulin signaling experiments, tissues were collected 15 min after an intraperitoneal injection of insulin in fasted mice as previously described (10).

### mRNA quantification by real-time PCR

Total RNA was isolated using the RNeasy kit (Qiagen) according to the manufacturer’s protocol, as previously described (10). Using a High-Capacity cDNA Reverse Transcription Kit (Applied Biosystems), total RNA was reverse transcribed to obtain cDNA. Quantitative real time-PCR was performed using Fast SYBR Green PCR Master Mix (Applied Biosystems) in the AB7900 HT Fast Real-Time PCR System. Data analysis was performed using the DDC(T) method and normalized to 18S, unless otherwise indicated. All mouse primers were purchased from McMaster University’s MOBIX Lab.

For the quantitative real-time PCR, the following human primer forward (F) and reverse (R) sequences were used: FAS, F:5′AAGTTGCCCGAGTCAGAGAA-3′, R:5′TGAGGCTGGGTTGATACCTC-3′; 18S, F:5′AGTCCCTGCCCTTTGTACACA-3′, R:5′CGATCCGAGGGCCTCACTA -3′; diacylglycerol *O*-acyltransferase-2, F:5′TCTCAGCCCTCCAAGACA TC-3′, R:5′GCCAGCCAGGTGAAGTAGAG-3′; Scd-1, F:5′TGGGTTGGCTGCTTGTG -3′, R:5′GCGTGGGCAGGATGAAG -3′; SREBP-1, F:5′TGCTCCAGCTCATCAACAAC -3′, R:5′GGCCAGAGAAGCAGAAGAGA -3′; GFP, F:5′CATGGTCCTGCTGGAGTTCGTGAC -3′, R:5′CCATTATAAGCTGCAATAAACAAGTTAACAACAACAATTG -3′; ADRP, F: 5′AAGAGAAGCATCGGCTACGA -3′, R: 5′GGCGATAGCCAGAGTACGTG -3′; CPT1, F: 5′CAGAGGATGGACACTGTAAAGG -3′, R: 5′CGGCACTTCTTGATCAAGCC -3′; PPARa, F: 5′ATGCCAGTACTGCCGTTTTCA -3′, R: 5′GGGCCTTGACCTTGTTCATGT -3′; CPT2, F: 5′GGATAAACAGAATAAGCACACCA -3, R:5′GAAGGAACAAAGCGGATGAG -3′; SREBP2, F: 5′GTGGAGCAGTCTCAACGTCA -3′, R: 5′TGGTAGGTCTCACCCAGGAG -3′; LDLR, F: 5′GAGGAGCAGCCACATGGTAT-3′, R: 5′ GCTCGTCCTCTGTGGTCTTC -3′; PCSK9, F: 5′ TTGCAGCAGCTGGGAACTT -3′, R: 5′ CCGACTGTGATGACCTCTGGA -3; TDAG51, F: 5′CCGAGGAAGGGCTACTGCTCA-3′, R: 5′CTCAAGGCTGGCGACGGTGG-3′.

### Cell culture and treatment

Huh7 immortalized human hepatocyte cell line (American Type Culture Collectiion), free from *mycoplasma* contamination, were maintained in 5% CO_2_ at 37 °C and cultured in Dulbecco's Modified Eagle's Medium or Willams' E medium, supplemented with 10% v/v fetal bovine serum, 100 IU/ml penicillin, and 100 μg/ml streptomycin. Huh7 cells were authenticated by phenotypic methods, including cell morphology. For experimental procedures designed to examine the effect of TDAG51 on lipid accumulation, cells were transfected using XtremeGENE (Sigma–Aldrich) and either a GFP or TDAG51-GFP plasmid (54) for 24 h and then treated with agents known to stimulate lipid droplet accumulation, such as oleate (200 μM, Sigma–Aldrich) for an additional 24 h.

### Fluorescent imaging

Six-micrometer thick cryosections were sectioned at −20 °C, then fixed with cold methanol, washed with 70% ethanol, washed with water, stained with 4′,6-diamidino-2-phenylindole, then cover slipped with Permafluor (Thermo Fisher Scientific). GFP and 4′,6-diamidino-2-phenylindole fluorescence were visualized with a Zeiss AxioCam ICc3 Imager.Z1.

### Statistical analysis

All data are expressed as the mean ± SD. When two groups were analyzed, an unpaired, two-tailed Student *t* test was utilized unless otherwise indicated as a one-tailed Student *t* test. When comparing more than two groups, the one-way ANOVA was utilized. Data points from the same animals collected at different time points are analyzed by two-way ANOVA. Statistical significance was defined as *p* <0.05. Statistical analyses and graphs were performed and generated using Microsoft Excel (https://www.microsoft.com/en-ca/microsoft-365/excel) and PRISM (https://www.graphpad.com/features) software.

## Data availability

All relevant data is presented in the main manuscript and Supporting information file.

## Supporting information

This article contains supporting information.

## Conflict of interest

The authors declare that they have no conflicts of interest with the contents of this article.
