# Supplementary figures and images for: Restoration of the ER stress response protein TDAG51 in hepatocytes mitigates NAFLD in mice
Source: J Biol Chem. 2024 Jan 16;300(2):105655. doi: 10.1016/j.jbc.2024.105655 (PMC10875272; doi:10.1016/j.jbc.2024.105655)

**Yousof et al. 2023 Supplemental Figure 1**

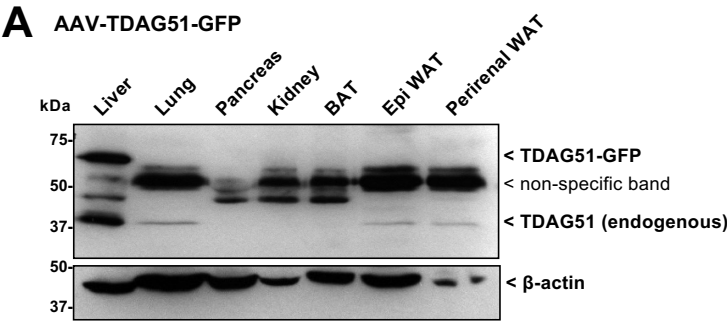

Supplement: Supporting Figure S1 [file mmc1.pdf]
