# Supplementary material for: Restoration of the ER stress response protein TDAG51 in hepatocytes mitigates NAFLD in mice
Source: J Biol Chem. 2024 Jan 16;300(2):105655. doi: 10.1016/j.jbc.2024.105655 (PMC10875272; doi:10.1016/j.jbc.2024.105655)
Supplement: Supporting Figure S1 legend [file mmc2.docx]

**Supplemental Figure 1. Liver-specific expression of TDAG51-GFP fusion protein in chow-fed wild-type (WT) mice. WT C57BL/6J mice 8 weeks of age were injected with AAV encoding TDAG51-GFP. After 4 weeks post-injection, tissues were immunoblotted.** *A,* The immunoblot shown in Figure 2A was stripped and re-probed with an anti-TDAG51 antibody to confirm the expression of TDAG51-GFP (68-kDa). TDAG51-GFP fusion protein was exclusively expressed in liver, compared to other tissues. β-actin image was used to demonstrate equivalent protein loading.
